# Sentiment Analysis of Animated Film Reviews Using Intelligent Machine Learning

**DOI:** 10.1155/2022/8517205

**Published:** 2022-07-20

**Authors:** Cheng Chen, Bin Xu, Jong-Hoon Yang, Mi Liu

**Affiliations:** Department of Digital Image in Sangmyung University, Seoul 03015, Republic of Korea

## Abstract

Film is an essential expression of a country's cultural soft power in terms of cross-cultural exchange. In addition, film is also the most direct and favourable means of communication. Along with the expansion and development of the Chinese film market, outstanding animation films have emerged in recent years. Animated films have both artistic and commercial properties and can not only have a cultural impact but can also contribute to economic growth. For this reason, our country is now paying more and more attention to the development of animated films. Specifically, animated films not only represent a country's cultural soft power and national image, but they are also a symbol of the strength of a country's cultural industry. As a reflection and extension of China's culture and ideology, animated films play an important role in enhancing cultural confidence and cultural export. In recent years, China's economy has shown a steady and sustained growth trend. At the same time, with the rapid development of internet technology, social networking has gradually penetrated into all aspects of people's lives. Various social networking forums, websites, and sites have emerged. While satisfying a wide range of needs, they also provide information on product reviews, social reviews, and service reviews. These reviews contain feedback from the reviewer about the subject of the review. Tapping into the emotions in these reviews can provide consumers with shopping references and help businesses to optimise their products and improve their business strategies. With the help of modern internet technology and information technology, the modern movie industry, such as Cat's Eye Movies and other internet entertainment service platforms, has developed a model of online ticketing, offline movie viewing, and online reviews and feedback. The content of the reviews on these movie websites fully reflects the attitudinal views of the movie-going community. These reviews play a decisive role in the box office and the further spread of culture. As a result, in order to better understand the audience's emotional tendencies and needs, it is necessary to carry out sentiment analysis and deep semantic mining of animated film reviews. As the evaluation of film works considers many factors and is complex and variable, the choice of model is crucial in the process of sentiment analysis. Machine learning models represented by deep neural networks are more tolerant of sentence noise and have strong information discrimination and feature self-learning capabilities. As a result, intelligent machine learning is more advantageous for sentiment classification tasks. This study is a combination of textual data mining and statistical analysis from the perspective of viewers' comments to study the online reviews of animation films from different countries. At the same time, this research hopes to uncover meaningful information from the film reviews and the gap between Chinese and other countries' animation films, in order to provide a little help for the rise of domestic animation films.

## 1. Introduction

Film is the most direct means of transmitting a country's values, cultural spirit, and aesthetic pursuits in the context of international cultural exchange [1]. As a reflection and extension of a country's culture and ideology, cinema can enhance cultural confidence and plays a vital role in cultural export [2]. While American cinema still dominates the world, the Chinese film market has leapt to second place in terms of box office production, due to the rising consumption levels of the Chinese population, increasing urbanisation, and the demand for local cultural products. At the same time, the rapid development of computer technology [3] and information technology [4] has brought people into the age of interactivity. Users can freely edit and participate in submitting, publishing, and communicating rather than just browsing the web. As a result, the virtual online space has become a place where individuals can communicate and express their emotions [5]. More and more people tend to actively post their own comments about an event or product on social networks, thus expressing important information such as their own opinions, emotions, attitudes, and positions [6]. Together, this information becomes valuable textual data. With the expansion and development of China's film market and the deep involvement of mobile internet technology, the modern film industry, such as Cat's Eye Movies and other entertainment service platforms that integrate online movie ticketing, movie information, and fan interaction, has formed a model of online ticketing, offline movie viewing, and online evaluation feedback. These online reviews are contributed by users themselves and fully reflect their attitudes and opinions [7]. These reviews are numerous, open, and multidimensional and of high value density [8]. In addition, the reviews not only reflect the main ideas of the viewers and their likes and dislikes of the film but also have a subjective influence on nonviewers, thus changing the direction of the box office and playing a decisive role in the further spread of culture.

**Figure 1:**
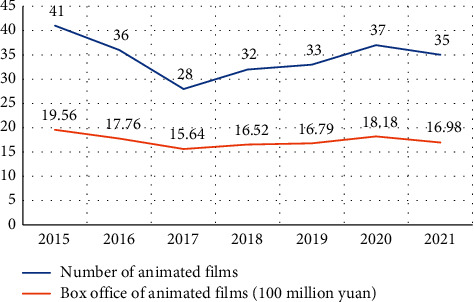
Data related to animation film industry from 2015 to 2021 in China.

Animation, which can reflect reality in a subjective form, is the form of cinema closest to people's imaginary worlds and is undergoing radical change. The arrival of digital technology has changed the relationship between animation audiences and animation to a great extent and has influenced the production and production of animation on a global scale [9, 10]. The modes of creation and distribution of Chinese animated films are also being experimented with and optimised [11]. The data related to the domestic animation film industry from 2015 to 2021 is shown in Figure 1. On the one hand, animated films bring economic benefits, and on the other hand, they represent the economic and cultural development of a country and its image to a certain extent [12]. Therefore, more and more experts and scholars are devoted to the study of animated films. The history of American and Japanese animated films has been studied in China, and they are also in the process of learning how to move forward [13]. Japanese animation was also based on imitation of American animation and gradually found its own style of drawing [14]. When animation reached a certain level of development, scholars and experts shifted from studying animated films to studying the animation industry. In the US, the animation industry is based on animated films, while in Japan it is based on the production committee model [15]. China, however, is still in its infancy and has not yet formed a complete industry chain. In the future, we can draw on the experience of other countries and combine it with our own development characteristics to gradually improve the animation industry. Either way, we need a sound mechanism for the protection of intellectual property rights, a reasonable division of labour, advanced technology, and a mechanism for the training of talents [16]. In addition, with the relevant policy guidance and support, there is a need to actively develop animation-related derivatives. After all, animation is not only an art form but also a commodity, with value added in all its aspects [17].

In addition to reflecting the thoughts and emotions of filmgoers, film criticism is also a good way of reflecting the views of the viewing public on a film as a whole, thus promoting the development of the film industry [18, 19]. At this stage, the Chinese film industry is undergoing structural adjustment. In an era of high-quality development, the pursuit of high box office sales must be accompanied by a focus on quality [20]. The film industry is now being restructured. Therefore, the analysis of film reviews has the following practical implications. First, it can provide producers with ideas for producing films that better meet the needs of the public [8]. Second, it can help cinemas to set up their schedules and promote their reviews [21]. In addition, it can meet the cultural needs of the audience and further promote domestic consumption. Finally, it can use emotion analysis techniques to extract, analyse, and summarise the subjective information in the text, so as to make suggestions for the production of films with Chinese cultural characteristics [22].

Text mining has been studied abroad since the last century, and today, there is a mature theoretical foundation and technical framework [23]. In the course of its development, text mining has become more and more involved in various fields [24, 25]. Nowadays, the huge amount of web data generated every day has led more and more Chinese scholars to start research in this area, including more and more research in the area of textual criticism. Of course, scholars have also adopted many research approaches to film criticism [26]. With the continuous development of Chinese web information, automatic sentiment analysis of Chinese web reviews has become an urgent problem. Some research studies on film data are based on traditional statistical modelling ideas, while others combine big data with information extraction and visualisation of the massive amount of subjective review data obtained [27]. Their diverse approach to film data allows us to analyse film data from a wider range of perspectives. However, there is still a gap in the analysis of animation film reviews, and only by combining big data with adequate research on animation film reviews can we provide powerful recommendations to the animation film industry and provide progressive data support for the development of domestic animation films [28].

Sentiment analysis is the process of analysing textual comments with emotional content by applying certain rules [29]. Sentiment analysis has important applications in areas such as online opinion monitoring, product word-of-mouth analysis, and market sentiment analysis. At present, there are three main types of sentiment analysis methods: sentiment dictionary and rule matching [30], traditional machine learning [31, 32], and deep learning [33]. The sentiment lexicon and rule matching methods depend heavily on the design of the lexicon and template rules. Traditional machine learning methods mainly analyse the syntactic and contextual features of sentences to classify sentiment [34]. However, the training of the models relies heavily on high-quality feature construction and selection and requires a large amount of human effort in the data labelling process. Deep learning is an algorithm based on deep neural networks, which is able to find and select effective features and has powerful discriminative and self-learning capabilities [35]. This allows deeper mining of semantic information in sentences, local feature abstraction, and memory, which is an advantage in sentiment classification tasks.

With the continuous development of information technology, the Internet and people's lives are becoming more and more inseparable. More and more users are surfing the Internet anytime and anywhere. Some people post their daily lives on social networking software, while others express their views and opinions on certain films and TV shows on entertainment websites. Underneath all this data, the seemingly irregular and trivial information has great value for research. This study uses web crawling technology to capture and analyse animated film review data. By analysing the reviews of different animated films by Chinese audiences, the study aims to assess the current development of Chinese animated films from an objective perspective. By further digging into the information behind the reviews, we will analyse the bottlenecks that restrict the development of Chinese animation films from different perspectives and how to break the barriers to achieve a strong animation nation sooner rather than later.

## 2. Data Processing for Animated Films

In today's internet era of big data, data are being generated all the time. More and more companies are now using big data technology to analyse user behaviour. For video websites, they recommend videos of interest to users based on their browsing, viewing, and favouriting behaviour. In the case of e-commerce platforms, they use information about customers' browsing, favourites, add-ons, purchases, and spending amounts to recommend the right products to users. This section focuses on the collection and processing of data.

### 2.1. Data Collection

**Figure 2:**
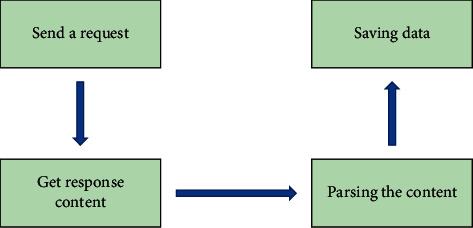
Basic process of crawling.

Python is an object-oriented interpreted computer programming language with a clear and concise syntax and a rich and powerful library, and the Python crawler is a program that automatically crawls the open domains of the Internet for data that is valuable to us. The crawler architecture consists of a scheduler, a URL manager, a web page downloader, a web page parser, and an application, and the general steps are shown in Figure 2.

**Figure 3:**
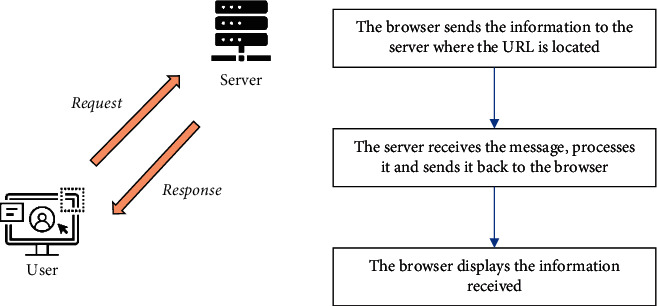
Process of request and response.

The request and response when crawling is shown in Figure 3. First, a request is made to the target site via the https library, that is, a request is sent, and then a response from the server is awaited. Then, if the server responds properly, it gets a response. The content of the response is the content of the page to be retrieved, which may be of a type of HTML, Json string, binary data, etc. The HTML type obtained is parsed using regular expressions and web parsing libraries. Finally, the data can be saved as text, to a database or in a specific format.

### 2.2. Split-Word Processing

Word segmentation is the process of dividing a given text into lexical units. Western languages such as English use spaces as word separators, so naturally words can be separated according to spaces. Chinese is not written with word separators, so it is necessary to first use a word separation algorithm. Chinese word segmentation is the first part of sentiment analysis, and if it is not carried out correctly, the value of the subsequent operations will be reduced. The basic approaches to word segmentation include dictionary-based, statistical and comprehension-based approaches.

The lexicon-based approach is also known as rule-based lexicography. This method is essentially string matching, where a lexicon is manually created and matched and sliced in a certain way. These algorithms are coarse classification models, which are simple and efficient to implement, but rely heavily on dictionaries and are not effective in dealing with ambiguities and unregistered words. Comprehension-based word separation is the use of computers to simulate the human mind to achieve the effect of word separation in sentences. The basic idea is to use syntactic and semantic analysis in the separation of Chinese words. Due to the semantic complexity of the Chinese language, it is difficult to compile all types of language into a form that can be read directly by machines. As a result, such systems are still not yet available.

### 2.3. Text Vocabulary Vectorisation

The number of comments freely edited by different users is enormous and highly unstructured. There are no structured or normalised grammars and patterns that can be processed and analysed using off-the-shelf mathematical or statistical models. Therefore, it is first necessary to translate real text into a representation that can be easily processed by machine learning algorithms. Words are the most basic unit of text, and the textual vocabulary in a comment is transformed into a vector of real numbers or other forms, and the formal representation is used to train and make decisions about the learning model. In order to classify textual sentiment at coarse granularity and textual sentiment at fine granularity, Word2Vec is applied for word vectorisation at coarse granularity.

**Figure 4:**
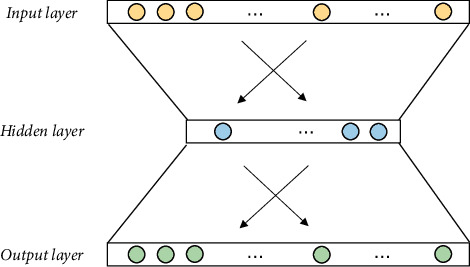
Neural network structure of the Word2Vec model.

The Word2Vec model can cope with the problems of vector sparsity and lack of semantic information that arise when using one-hot encoding for word vectorisation. As a result, the representation of word vectors can be simplified, and word vectors with similar semantics can be placed relatively close to each other in space. The Word2Vec model is structured with a text body as input and a vector space as output. The word vector generated by the model is a low-dimensional spatial vector, which allows the data to be compressed in size while capturing contextual information. The input layer is a one-hot vector of contextual words, and the hidden layer has no activation function, that is, a linear unit. The neural network structure of the Word2Vec model is shown in Figure 4.

As can be seen in Figure 4, the Word2Vec word vectorisation tool is essentially a three-layer neural network model—an input layer, a hidden layer, and an output layer. The method can map the original vector space of words to a new vector space, and words with similar semantics are mapped to similar positions in the new vector space, representing the semantics of the text in a distributed numerical form. In addition, the word vectors facilitate clustering analysis, using cosine similarity or Euclidean distance to find two words with similar meanings. The Word2Vec model is not a monolithic model but is made up of several models, including CBOW and Skip-Gram, two shallow word vector training models that have been commonly used in the field of natural language processing. Both models use artificial neural networks as classification algorithms. After training a random *N*-dimensional vector for each word, the optimal vector for each word is obtained using CBOW or Skip-Gram.

The CBOW model assumes that the current word is predicted based on context. Since the CBOW model has multiple contextual words, we average these word vectors. Given any m-tuple in the training corpus, the window size is *W*. Thus, the output is a weighted average of the input word vectors:

(1) $$\begin{array}{c} p=\frac{1}{2W}\sum_{l-W\leq j\leq l+W}e\left(\omega_{j}\right), \end{array}$$

where *p* refers to the probability of predicting the central target word.

The word vector *V* is the only neural network parameter in the CBOW model. Optimize the word vector matrix *M* to maximize the log-likelihood of all words:

(2) $$\begin{array}{c} M^{*}=\underset{M}{\arg\text{max}}\sum_{\omega_{i}}\text{log}p. \end{array}$$

## 3. Neural Network Structures for Sentiment Analysis

### 3.1. Long Short-Term Memory Network

Long short-term memory networks (LSTM) are improved from recurrent neural networks (RNN), which are a type of neural network structure specialising in the accurate modelling of temporal data. The introduction of mechanisms such as parameter sharing and long-term dependencies has improved its performance in sequential problems compared to traditional fully connected networks. In the case of sequential modelling of utterances, for example, a fully connected network trains a unique parameter for each input feature to obtain linguistic rules for each position in the sentence. The RNN, on the other hand, shares the same parameters at a specific time step and will also provide information from several previous time points for the prediction of the current time point through a corresponding memory mechanism.

**Figure 5:**
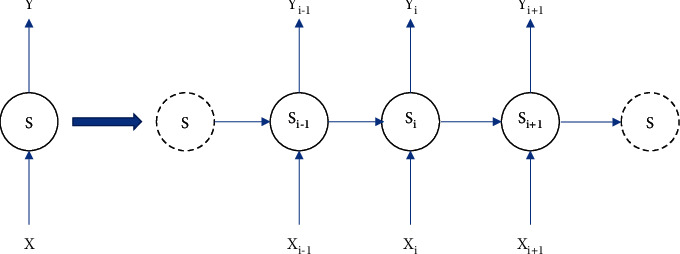
Classical network structure of RNN.

Comments made by users on social media platforms are typically sequential data, where each word is dependent on the previous word. Therefore, when analysing a comment, it is not enough to understand each word in a sentence in isolation. RNN contains time-series memory and is suitable for this type of modelling problem as they are capable of mining the text for both temporal and semantic information. The classical network structure is shown in Figure 5, with the network structure running in a temporal loop on the left and the network structure expanded in temporal order on the right.

In RNN, the dependencies in the sequence can be expressed as

(3) $$\begin{array}{c} S_{i}=\sigma \left(VX_{i}+US_{i-1}+a\right), \end{array}$$

where *S*_*i*_ refers to the hidden state at time *i*, *σ* refers to the tanh function, *V* denotes the weight matrix of the output from the hidden layer to the output layer, and *a* indicates the linear relationship.

However, when learning a time series of arbitrary length, RNN's ability to perceive information from long back decreases as the sequence interval increases, resulting in long-term dependencies and gradient disappearance. As a result, it is difficult to capture long-distance dependencies and cannot effectively link historical information together, resulting in large errors in the sentiment analysis of utterances.

**Figure 6:**
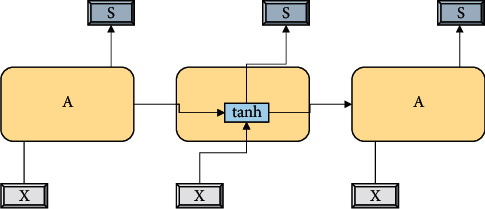
Structure of the LSTM.

The LSTM structure adds a memory cell state to the RNN hidden layer structure. At the same time, the LSTM structure uses three gating mechanisms to control the retention information of the previous time step, the input information of the current time step, and the output information of the current time step. The cell state changes over time to transmit the model's memory information. The forgetting gate is used to selectively forget previously outdated state information, acting on the memory cell state and selectively forgetting information in the memory cell. The input gate is used to decide which information to include in the input state at the current time point. Output gates are used to obtain the output information for the current time node. The combination of the memory cells and these control gates avoids the problem of gradient disappearance or gradient explosion and enhances the ability to process long sequences of data. The structure of the LSTM is shown in Figure 6.

In the LSTM structure, we have

(4) $$\begin{array}{c} S_{i}=E\left(X_{i},S_{i-1}\right), \end{array}$$

where *S*_*i*_ refers to the hidden state at time *i* and *E* is the following functions:

(5) $$\begin{array}{c} e_{i}=\sigma \left(V_{e}X_{i}+V_{e}S_{i-1}+a_{e}\right),\\ z_{i}=\sigma \left(V_{i}X_{i}+V_{i}S_{i-1}+a_{i}\right), \end{array}$$

where *V*_*e*_ and *V*_*i*_ are different parameters corresponding to the state.

### 3.2. Model Assessment

**Figure 7:**
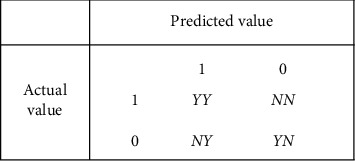
Confusion matrix.

Once the results of the classifier have been obtained, it is also important to evaluate how well the classifier performs. The most common metrics used to evaluate the performance of classifiers are accuracy, precision, recall, *F*-value, and ROC. The confusion matrix is often used to evaluate the accuracy of the classification results during the algorithm learning process. The confusion matrix, also known as the error matrix, is a standard format for representing accuracy evaluation, as shown in Figure 7.

Since the confusion matrix can quickly help one to analyse the misclassification of each category, it is easy to adjust the model analytically. In the confusion matrix, the rows represent the true observations and the columns represent the inferred results of the classifier.

Accuracy refers to the probability that the correct outcome is predicted as a percentage of the total sample and is given by the formula:

(6) $$\begin{array}{c} \text{accuracy}=\frac{YY+YN}{YY+NY+NN+YN}. \end{array}$$

The precision is the probability that a sample is actually positive out of all the samples that are predicted to be positive, and the expression is

(7) $$\begin{array}{c} \text{precision}=\frac{YY}{YY+NY}. \end{array}$$

Recall is the probability of a positive sample being predicted out of an actual positive sample and the expression is

(8) $$\begin{array}{c} \text{recall}=\frac{YY}{YY+NN}. \end{array}$$

By constructing a sentiment dictionary for the film domain and using the sentiment analysis algorithm described above, binary classification was performed on a film review dataset. To demonstrate the validity of the model, two other methods were used for comparison. The first method uses a sentiment dictionary on the Internet that does not introduce sentiment words in the film domain to analyse the sentiment polarity of the review data. The second method is a sentiment analysis of the review data using the SnowNLP package in Python. For ease of notation, the methods before and after the extension of the sentiment dictionary are named *P* and *Q*, respectively, and the experimental results are shown in Table 1.

Therefore, the use of the expanded sentiment dictionary in the film domain for sentiment polarity analysis significantly improved the classification results. This indicates that the emotion calculation rules designed in this study are reasonable and the emotion lexicon constructed in the film domain is effective. However, there are limitations in this approach, as it does not consider the complexity of Chinese semantic expressions and the limited dataset, and the classification results are still not ideal.

## 4. Conclusion

Film is not just an entertainment industry, it reflects life, and art comes from life but is above it. Film conveys social values and concepts to the audience, and while the economy is growing, spiritual progress is also a reflection of social progress. The core of a good animation work is of course a good story, and it is inseparable from the production team behind the scenes, behind every excellent work there are countless people's dedication and efforts, and each frame is a cohesion of their hearts and souls. In this age of diversity, there is also a need for cultural diversity to meet the demand. There may not be a single production that is universally popular, but every production has its own merits.

This paper focuses on intelligent machine learning-based text sentiment classification models, constructing several deep learning models and comparing their experimental results and methods. Further analysis is carried out based on the sentiment classification results. In terms of the selection of the dataset, the crawler is designed to crawl different animated movie reviews to ensure the timeliness of the data. The data were deduplicated, denoised, and then processed by word separation, while the reliability of the data is explored by multidimensional visualisation. In terms of training models and prediction methods, the paper constructs positive and negative sentiment labels for high and low scoring comments and selects the middle scoring bands as the candidate prediction dataset.

The work on the affective analysis of film criticism in this paper has yielded some results, but further research is needed before the research study can be further improved. Specifically, only the extraction of explicit evaluation objects has been completed in this paper. The extraction of implicit evaluation objects is less involved, and there is a need to improve the extraction of implicit attributes of evaluation objects in the future. In addition, this study only categorised the overall sentiment of the review data and failed to analyse the sentiment of each sentence in terms of cinema, plot, and special effects. Future research could be based on aspect-based sentiment analysis for further exploration.

## Figures and Tables

**Table 1:** Comparison between different models.

Model	Accuracy (%)	Precision (%)	Recall (%)
SnowNLP	69.27	64.45	59.97
Model *P*	77.46	74.21	66.34
Model *Q*	81.63	79.06	74.53

## Data Availability

The labelled dataset used to support the findings of this study is available from the corresponding author upon request.

## References

[1] Vereș S., Magdaș I. (2020). The use of the educational animated film in primary education in Romania. Literature review. *Romanian Review of Geographical Education*.

[2] Biltereyst D., Meers P. (2018). Film, cinema and reception studies. *Reception Studies and Audiovisual Translation*.

[3] Cheng B., Fan C., Fu H., Huang J., Chen H., Luo X. (2022). Measuring and computing cognitive statuses of construction workers based on electroencephalogram: a critical review. *IEEE Transactions on Computational Social Systems*.

[4] Salahshour Rad M., Nilashi M., Mohamed Dahlan H. (2018). Information technology adoption: a review of the literature and classification. *Universal Access in the Information Society*.

[5] Vlachopoulos D., Makri A. (2019). Online communication and interaction in distance higher education: a framework study of good practice. *International Review of Education*.

[6] Robinson H. A., Kilgore W., Warren S. J. (2017). Care, communication, support: core for designing meaningful online collaborative learning. *Online Learning*.

[7] Ma H., Kim J. M., Lee E. (2019). Analyzing dynamic review manipulation and its impact on movie box office revenue. *Electronic Commerce Research and Applications*.

[8] Larasati U. I., Muslim M. A., Arifudin R., Alamsyah A. (2019). Improve the accuracy of support vector machine using chi square statistic and term frequency inverse document frequency on movie review sentiment analysis. *Scientific Journal of Informatics*.

[9] Azzajjad M. F., Tendrita M., Ahmar D. S. (2021). Effect of animation and review video making (arvima) in non-classical learning model on independent learning and students’ learning outcome. *Linguistics and Culture Review*.

[10] Wang H., Sharma A., Shabaz M. (2022). Research on digital media animation control technology based on recurrent neural network using speech technology. *International Journal of System Assurance Engineering and Management*.

[11] Lukyanova V., Koloskova O. (2020). Pragmatic potential of onomatopoeia in animated movies for children. *Global Journal of Foreign Language Teaching*.

[12] Mongar D. S., Chalermnirundorn N. (2020). The use of animated movies to enhance narrative writing skills of grade six bhutanese ESL Students. *Academic Journal Phranakhon Rajabhat University*.

[13] Pellitteri M. (2021). The European experience with Japanese animation, and what it can reveal about the transnational appeal of anime. *Asian Journal of Communication*.

[14] Alsubaie S. S., Alabbad A. M. (2020). The effect of Japanese animation series on informal third language acquisition among Arabic native speakers. *English Language Teaching*.

[15] Lamarre T. (2017). The animation of China: an interim report. *Journal of Chinese Cinemas*.

[16] Pei Y. S., Meng H. Y., Hou S. S., Bae K. H. (2018). The study on the promotion of Chinese animation industry-A Comparison of Korea. China and Japan. *The Journal of the Korea Contents Association*.

[17] Cui J. (2021). The pursuit of a national style of Chinese animation. *Animation: Critical and Primary Sources*.

[18] Hassan F. (2021). Philosophy leaves the movie theater: or, Stanley Cavell at the doors of a discipline. *Journal of Comparative Literature and Aesthetics*.

[19] Heriyadi N., Diana E. (2020). An analysis of social deixis in the Dressmaker movie. *Literary Criticism*.

[20] Zhou Y., Zhang L., Yi Z. (2019). Predicting movie box-office revenues using deep neural networks. *Neural Computing & Applications*.

[21] Lee J. H., Jung S. H., Park J. (2017). The role of entropy of review text sentiments on online WOM and movie box office sales. *Electronic Commerce Research and Applications*.

[22] Tarigan Z. J. H., Basuki R., Siagian H. (2020). The impact of information technology quality on electronic customer satisfaction in movie industry. *International Journal of Data and Network Science*.

[23] Kumar S., Kar A. K., Ilavarasan P. V. (2021). Applications of text mining in services management: a systematic literature review. *International Journal of Information Management Data Insights*.

[24] Kobayashi V. B., Mol S. T., Berkers H. A., Kismihók G., Den Hartog D. N. (2018). Text mining in organizational research. *Organizational Research Methods*.

[25] Hyndman B., Pill S. (2018). What’s in a concept? A Leximancer text mining analysis of physical literacy across the international literature. *European Physical Education Review*.

[26] Dutta A., Bhattacharjee B., Sridhar A. (2017). Identifying the causal relationship between social media content of a Bollywood movie and its box-office success – a text mining approach. *International Journal of Business Information Systems*.

[27] Singh V., Saxena P., Singh S., Rajendran S. (2017). Opinion mining and analysis of movie reviews. *Indian Journal of Science and Technology*.

[28] Novendri R., Callista A. S., Pratama D. N., Puspita C. E. (2020). Sentiment analysis of YouTube movie trailer comments using naïve bayes. *Bulletin of Computer Science and Electrical Engineering*.

[29] Yadav A., Vishwakarma D. K. (2020). Sentiment analysis using deep learning architectures: a review. *Artificial Intelligence Review*.

[30] Xu G., Yu Z., Yao H., Li F., Meng Y., Wu X. (2019). Chinese text sentiment analysis based on extended sentiment dictionary. *IEEE Access*.

[31] Cheng B., Lu K., Li J., Chen H., Luo X., Shafique M. (2022). Comprehensive assessment of embodied environmental impacts of buildings using normalized environmental impact factors. *Journal of Cleaner Production*.

[32] Cheng B., Huang J., Li J., Chen S., Chen H. (2022). Improving contractors’ participation of resource utilization in construction and demolition waste through government incentives and punishments. *Environmental Management*.

[33] Gan M., Cui H. (2021). Exploring user movie interest space: a deep learning based dynamic recommendation model. *Expert Systems with Applications*.

[34] Qian Y., Chen S., Li J. (2020). A decision-making model using machine learning for improving dispatching efficiency in Chengdu Shuangliu airport. *Complexity*.

[35] Da’u A., Salim N. (2020). Recommendation system based on deep learning methods: a systematic review and new directions. *Artificial Intelligence Review*.

